# Exposure Effects of Environmentally Relevant Concentrations of the Tricyclic Antidepressant Amitriptyline in Early Life Stage Zebrafish

**DOI:** 10.1021/acs.est.3c08126

**Published:** 2024-07-17

**Authors:** Sophie L. Gould, Matthew J. Winter, Maciej Trznadel, Anke Lange, Charles M. Hamilton, Rebekah J. Boreham, Malcolm J. Hetheridge, Andrew Young, William H. J. Norton, Charles R. Tyler

**Affiliations:** † Biosciences, Faculty of Health and Life Sciences, University of Exeter, Stocker Road, Exeter, Devon EX4 4QD, U.K.; ‡ Department of Genetics and Genome Biology, College of Life Sciences, University of Leicester, University Rd., Leicester LE1 7RH, U.K.

**Keywords:** ecotoxicology, antidepressant, pharmaceuticals, behavior, bioaccumulation

## Abstract

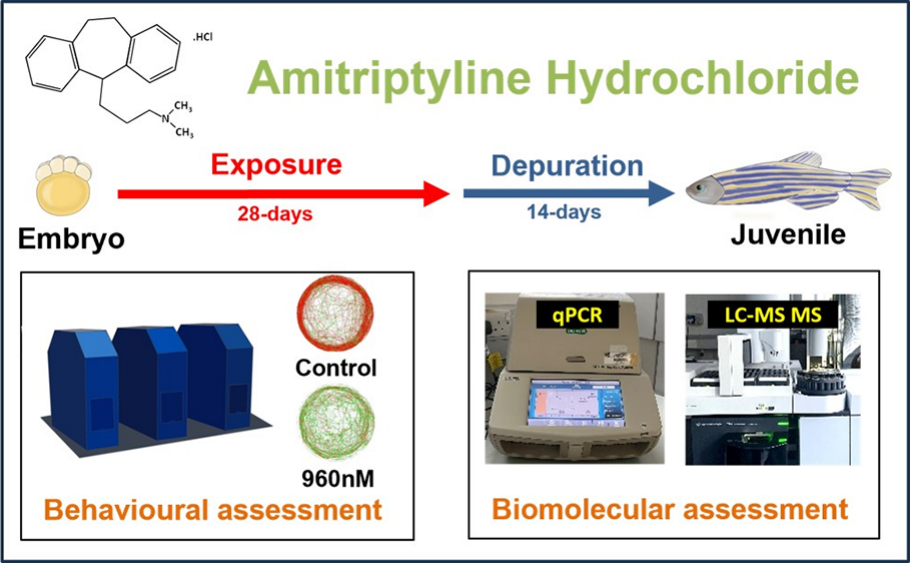

Antidepressants are one of the most globally prescribed classes of pharmaceuticals, and drug target conservation across phyla means that nontarget organisms may be at risk from the effects of exposure. Here, we address the knowledge gap for the effects of chronic exposure (28 days) to the tricyclic antidepressant amitriptyline (AMI) on fish, including for concentrations with environmental relevance, using zebrafish (*Danio rerio*) as our experimental model. AMI was found to bioconcentrate in zebrafish, was readily transformed to its major active metabolite nortriptyline, and induced a pharmacological effect (downregulation of the gene encoding the serotonin transporter; *slc6a4a*) at environmentally relevant concentrations (0.03 μg/L and above). Exposures to AMI at higher concentrations accelerated the hatch rate and reduced locomotor activity, the latter of which was abolished after a 14 day period of depuration. The lack of any response on the features of physiology and behavior we measured at concentrations found in the environment would indicate that AMI poses a relatively low level of risk to fish populations. The pseudopersistence and likely presence of multiple drugs acting via the same mechanism of action, however, together with a global trend for increased prescription rates, mean that this risk may be underestimated using current ecotoxicological assessment paradigms.

## Abstract 2

Antidepressants are widely detected in the aquatic environment and can affect wild fish physiology and behavior. We show that amitriptyline bioconcentrates in fish tissues with downstream pharmacological effects.

## 1 Introduction

Anxiety and depression are now the most frequently diagnosed psychiatric conditions. For example, in 2017/18, almost one-fifth of England’s adult population (7.3 million) received medical treatment to manage symptoms of these disorders.^1^ Antidepressant drugs are the primary treatment for these conditions, and globally, they are one of the most commonly prescribed classes of drug (e.g., >80 million prescriptions dispensed annually in England).^2^ Of these drugs, the tricyclic antidepressant amitriptyline (AMI) is the most prescribed in England (by weight, 11.2 tons per year) and one of the most prescribed in the USA (at 23.1 tons per year).^200,201,202^ Reflecting this, AMI is also one of the most frequently detected human drugs in the aquatic environment.^3^ It is typically detected at ng/L to low μg/L levels in wastewater treatment plant (WWTP) effluents and surface waters,^4^ but has been detected at concentrations as high as 196 ng/L (in the Atibaia’s River basin, Brazil).^5^ Importantly, the major metabolite of AMI, nortriptyline (NOR), is also biologically active and is itself also prescribed for depression.^6^ Despite this high usage and widespread detection, the vast majority of ecotoxicological data have been generated on the Selective Serotonin Reuptake Inhibitors or SSRIs, a related group of serotonin transporter (SERT)-selective antidepressants clinically favored due to a lower rate of side effects.^4,7^ Consequently, our knowledge of the potential environmental impact of AMI is limited.

The primary therapeutic mechanism of action of both AMI and NOR is the inhibition of the noradrenaline (or norepinephrine, NE) and serotonin (5-hydroxytryptamine, 5-HT) transporters (SLC6A2 and SLC6A4, NET and SERT, respectively), which serves to elevate local NE and 5-HT concentrations by decreasing their reuptake from the synaptic cleft (reviewed in ref (8)). Importantly, these drug targets are highly conserved across diverse taxonomic groups^9,10^ meaning that wildlife species, including fish, may be susceptible to the effects of these drugs when they enter the aquatic ecosystem following patient use and excretion. Fish are potentially especially vulnerable to the neurobehavioral effects of antidepressants, as in addition to showing considerable target conservation for these with humans; these drugs are also readily uptaken from the water via the gills.^11^ As such, the effects of antidepressants in fish are receiving increasing attention (e.g., refs (12−15)). However, studies on the effects of AMI specifically are still relatively limited and focused on pharmacological or toxicological impacts of exposure (e.g., refs (16−18)). It has been reported, however, that exposure to 0.01 μg AMI/L, a level detected in some surface waters, accelerates hatching rates in zebrafish (*Danio rerio*),^19^ and an exposure to 0.2 ug/L AMI for 7 days in the gilt-head bream (*Sparus aurata*) was shown to alter the profile of metabolites in both the brain and liver, indicating significant metabolic perturbations.^17^

Here, we assessed the effects of AMI under chronic (28 day) exposure conditions in early life stage zebrafish, incorporating a depuration period of 14 days in clean water to analyze for persistence. The monoaminergic circuitry develops very early on in the development of the zebrafish (e.g., refs (20−22)) and zebrafish embryos and larvae have been shown previously to be responsive to the pharmacological effects of antidepressants (e.g., refs (23) and (24)). Early life stage fish may also be both more susceptible to drug uptake from the water environment due to their relatively high surface area to volume ratio and less developed metabolic capability compared with older animals (e.g., ref (25)). The end points we selected for assessment were those most likely to be affected based on the mechanism of action of AMI including brain monoamine levels, target gene expression, and anxiolytic behaviors.

## 2 Materials and Methods

### 2.1 Selection and Preparation of AMI Test Solution

The experimental design used was based upon OECD guideline 210 with minor adjustments.^26^ AMI hydrochloride (AMI, CAS number 549-18-8; ≥98% purity) was obtained from Sigma-Aldrich (St. Louis, MO, USA). Nominal exposure concentrations of AMI were 0 nM, 0.0096 nM (0.003 μg/L), 0.096 nM (0.03 μg/L), 0.96 nM (0.3 μg/L), 9.6 nM (3 μg/L), 96 nM (30 μg/L), and 960 nM (300 μg/L), selected to capture the lower and upper limits of the human therapeutic range (50–300 μg/L,^27^ and reported environmental levels in effluent (0.015–0.227 μg/L) and surface water (0.012–0.070 μg/L) (e.g., see ref (4)).

Stock exposure solutions were prepared in embryo-larval culture water, which consisted of mains tap water filtered by reverse osmosis and then reconstituted with Analar-grade mineral salts to a standard synthetic freshwater composition (final ion concentrations: 117 mg/L CaCl_2_.2H_2_0, 25.0 mg/L NaHCO_3_, 50 mg/L MgSO_4_.7H_2_0, 2.3 mg/L KCl, and 1.25 mg/L tropic marine sea salt, giving a conductivity of 300 mS/m).

### 2.2 Zebrafish Brood Stock Housing and Maintenance

Adult zebrafish of Wild Indian Karyotype (WIK) were supplied by the University of Exeter Aquatic Resource Centre and reared under optimal conditions for spawning (28± 1 °C; 12 h light: 12 h dark cycle, with 20 min dusk–dawn transition periods). Water was routinely monitored for temperature, pH, conductivity, ammonia, nitrite, and nitrate, all of which were maintained within appropriate limits for zebrafish. All work was undertaken under project and personal licenses granted by the UK Home Office under the UK Animals (Scientific Procedures) Act and approved by the University of Exeter’s Animal Welfare and Ethical Review Body.

### 2.3 Zebrafish Exposure to AMI

Embryos were collected from group-spawned adults shortly after lights on and assessed for successful fertilization and stage of development. Viable embryos were then pooled and transferred randomly to Petri dishes in groups of 90 (in clean water) before being transferred, in these same groups, into the test vessels approximately 1.5 h postfertilization (hpf). Embryos were exposed to AMI via a flow through system for 28 days (Supplementary Figure 1), as a chronic exposure scenario.^26^

**Figure 1:**
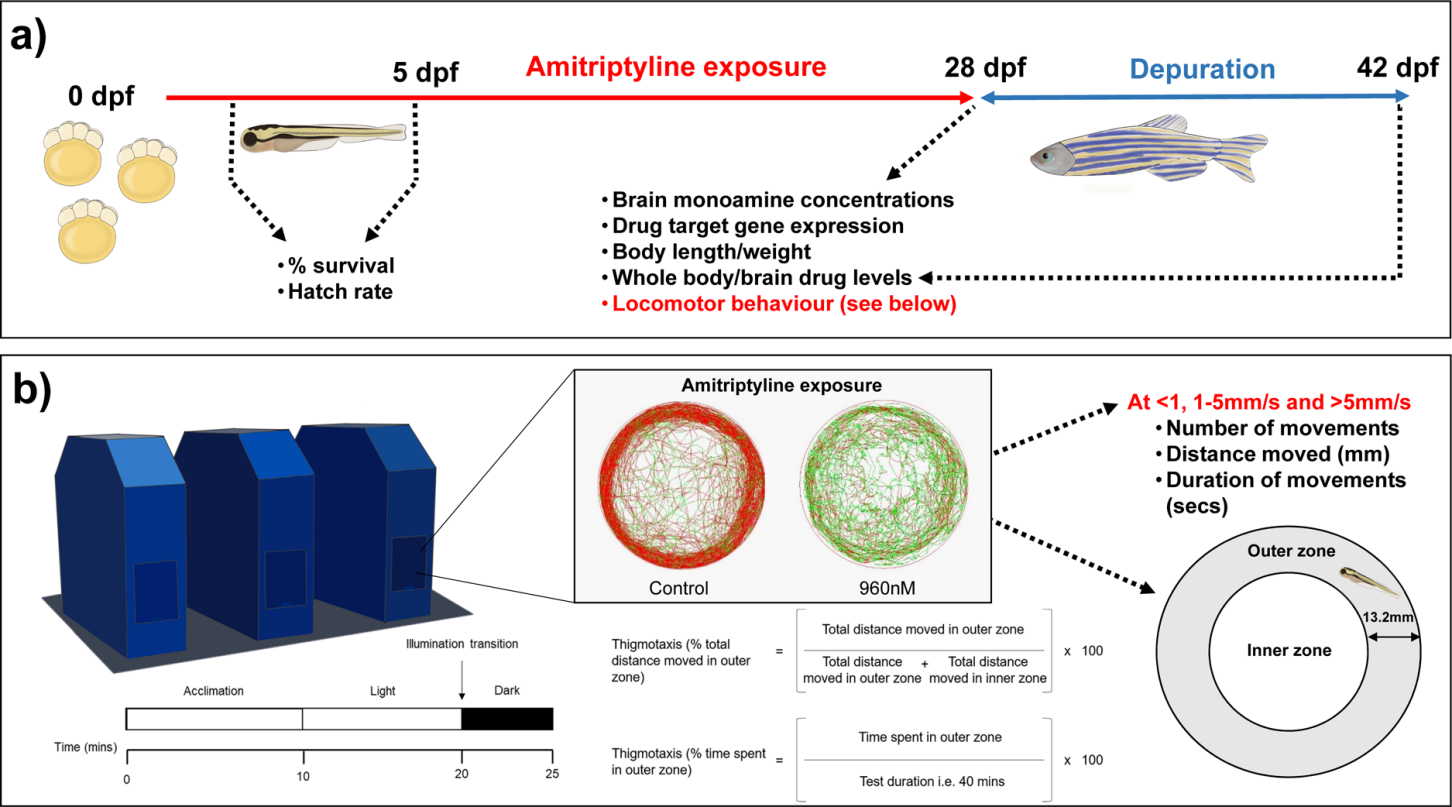
Schematic representation of AMI exposure experimental design and end points measured. (a) Illustrating the exposure protocol used along with the experimental timelines, along with the stages at which various end points were assessed. (b) Illustrating the protocol used for assessing zebrafish behaviors after 28 days of AMI exposure and after a further 14 days of depuration.

In addition to the exposure period, a 14 day depuration period was included to assess AMI elimination and recovery of any treatment induced effects. Fourteen days was selected based on the reported half-lives of AMI and fluoxetine (FLX) in humans (1–4 days and 20 h, respectively),^28−30^ compared to that reported for FLX in fish (<24 h for embryos and 9 days for adults),^31−34^ as there are no available data for AMI depuration in fish. Stock solutions and culture water were delivered to mixing chambers to achieve the desired nominal exposure concentrations and then fed into secondary vessels to ensure complete mixing before being delivered to the exposure tanks (5 replicate vessels per treatment, with 1 L tank volume; see Supplementary Table 1 for flow rates). pH, temperature, dissolved oxygen, and ammonia were also measured at least once per week during the experiment (see Supplementary Table 2). Stock solutions were replenished every 3 days, and dosing was initiated 2 weeks prior to the addition of animals, during which AMI concentrations were measured by LC-MSMS to ensure stable conditions were attained (see Supplementary Table 3). Water samples were also analyzed across the experimental duration to ensure that consistent exposure concentrations were maintained (see Supplementary Table 4). From 5 days postfertilization (dpf), larvae were fed at a rate of 300% of the average total body weight of comparably aged larvae with dry food (Zebrafeed, Sparos), and this ration was adjusted accordingly using the age-adjusted weight of fish lost, based upon the result of a preliminary feeding trial (see Supplementary Section 2). The basic experimental design and sampling points are listed in Figure 1.

### 2.4 Measurement of AMI in Water Samples

Water was sampled from each tank once a week (triplicate samples were taken for one replicate tank per treatment, alternating the tank at each time point) and analyzed in duplicate using a TSQ Vantage triple quadrupole mass spectrometer. Quantification was performed by Multiple Reaction Monitoring (MRM) of two characteristic transitions for AMI and one for the d_3_-AMI internal standard. A full outline of the methods used can be found in Supplementary Section 1.

### 2.5 Assessment of Apical End Points

Mortality and hatching rate were recorded daily and at 28 dpf, and after depuration at 42 dpf, fish were humanely terminated via anesthetic overdose and then photographed. From the photographs, fork length was measured using ImageJ. Wet weight was measured after the fish had been blotted dry, and fish were then snap frozen in liquid nitrogen and stored at −80 °C for further analysis.

### 2.6 Measurement of AMI in Fish Tissue

At 28 and 42 dpf, whole bodies (WB) and dissected whole heads (WH) of zebrafish were frozen in liquid nitrogen and stored at −80 °C until analysis. On the day of analysis, whole bodies were thawed on ice and weighed. For half of the decapitated WH, weights were taken prior to and after removal of the eyes (referred to as HNE, heads no eyes), and the remaining half were left intact and weighed. The eyes were removed as they have been shown to be a major sink for compound accumulation,^35,36^ and we, therefore, expected that HNE samples would likely be more representative of actual brain concentrations of AMI. Full details of the extraction procedure are detailed in Supplementary Section 3. Briefly, individual samples were added to a mix of acetonitrile, water, and AMI/NOR internal standard, before being homogenized. The supernatant was analyzed using the TSQ Vantage triple quadrupole mass spectrometer as detailed in Supplementary Section 1.

Using the measured water and tissue concentrations of AMI and NOR, various bioconcentration factors were calculated using the following equation:
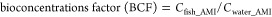

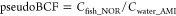
where *C*_fish_ is the concentration in the fish (mg kg^–1^, wet weight), *C*_water_ is the concentration in the water (mg L^–1^, nominal or measured), and pseudoBCF is the ratio of the NOR concentration in the fish and the AMI concentration in the test water.

### 2.7 Brain Monoamine Analysis via High-Performance Liquid Chromatography (HPLC)

At 28 and 42 dpf, fish from the control, 0.096 and 960 nM treatments (representing pharmacologically and environmentally relevant concentrations respectively) were terminated and their brains removed and analyzed for monoamine levels via HPLC using the method of Carreno Gutierrez et al. (2018).^37^ Neurotransmitters measured were: 5-HT, 5-hydroxyindoleacetic acid (5-HIAA, the main metabolite of 5-HT), norepinephrine (NE), dopamine (DA), 3,4-dihydroxyphenylacetic acid (DOPAC, the major metabolite of DA), and homovanillic acid (HVA, the product of degraded DA). The ratios of 5-HIAA/5-HT, DOPAC/DA and HVA/DA were calculated to assess monoamine turnover in the brain. A full outline of the methods used can be found in Supplementary Section 5.

### 2.8 5-HT Transporter/Receptor Gene Analysis Using qRT-PCR

To assess the impact of AMI exposure on modulation of the zebrafish serotonergic system, transcript levels of the main targets of AMI were measured using qRT-PCR. As a tertiary amine TCA, AMI is more potent as a modulator of 5-HT compared with NA and DA.^38^ Consequently, we opted to assess the modulation of the gene encoding the 5-HT transporter (SERT), specifically *slc6a4a*, as it is more widely expressed in the zebrafish brain compared with its paralogue *slc6a4b.*(20) In addition, we analyzed the zebrafish orthologue for *HTR1A*, namely, *htr1aa*, as its zebrafish paralogue *htr1ab* shows lower homology to human *HTR1A.*(20) The *htr2c* receptor gene was also selected to indicate altered levels of the zebrafish orthologue of human *HTR2C*.

From a subset of dissected brains stored at −80 °C, total RNA was extracted using the RNeasy Mini Kit (QIAGEN Ltd.) with an on-column DNase I digestion according to the manufacturer’s instructions. RNA concentration and purity were determined using a NanoDrop ND-1000 Spectrophotometer (Labtech). cDNA was synthesized from 1 μg of total RNA using random hexamers (Eurofins Genomcs, Germany) and M-MLV Reverse Transcriptase (Promega, UK) according to the manufacturer’s instructions. cDNA was stored at −20 °C for later use.

Target-specific qRT-PCR SybrGreen assays were optimized for each primer pair as described previously.^39^ Details of primer sequences and qPCR assay conditions are shown in Supplementary Section 6 and the Supporting Information, Table 8. qRT-PCR was performed on a CFX96 Real-time PCR System (Bio-Rad) using the Bio-Rad CFX Maestro Software Version 2.2 (Bio-Rad). PCR reactions were run in triplicate and each gene was processed in two PCR runs, each containing 28 samples alongside a no-template control (NTC) and positive control (PC, a pool of cDNA from 8 samples from different treatments). Efficiency (*E*)-corrected relative expression levels of target genes relative to a selected housekeeping gene (ribosomal protein L8; *rpl8*) were calculated based upon the arithmetic comparative method (2^–ΔΔ*Ct*^;^40^ with a correction for differences in *E* between the target and “housekeeping” gene.^41^

### 2.9 Assessment of Fish Behavior

To allow assessment of adequate numbers of animals from all treatments, behavioral assessments of individual fish were spread across exposure days 29 and 30, and depuration days 14 and 15, with fish from each treatment represented equally on each day (total *n* = 16). Assessment was undertaken in Petri dishes containing 50 mL of solution taken directly from the holding vessels to maintain exposure concentrations.

General locomotor activity, thigmotaxis, and light/dark responsiveness were quantified using the VideoTrack for Zebrafish videotracking system (software Version 2.5 with background subtraction, Viewpoint, France), equipped with infrared cameras.^42^ Animals were video recorded under infrared lighting for the dark phase and under white light (intensity ca. 500 Lux on the chamber stage) during the light phase. The use of a sudden lights on/off stimulus was incorporated to provide a mildly anxiogenic stimulus (e.g., Schnörr et al. 2012 aimed at improving sensitivity^43^). The rationale for this was that the therapeutic effects of AMI would likely be clearest under conditions of anxiety or stress, rather than under “normal” (stress free) conditions as previously suggested.

For general locomotion, the total number of movements, time spent moving, total distance moved, and average speed of movements were analyzed. The method used to assess thigmotaxis was based upon that of Schnörr *et al.*(43) Briefly, the Petri dish (total diameter 90 mm) was divided into a virtual outer zone (diameter 13.2 mm) and a virtual inner zone (diameter 63.6 mm), both with equal total areas of 3180.8 mm^2^. Both zones also had a width exceeding the average animal body length we measured at 10.86 ± 3.43 mm (*n* = 10). Data were retrieved for each parameter every 60 s and included the area of the whole test arena alongside the analysis of both the inner and outer zones separately. Thigmotaxis, defined as the proportion of time spent or distance moved in the outer zone, was calculated by using the equations outlined in Figure 1. For assessment, animals were left to acclimatize for 10 min followed by a 10 min period of lights on, and then an immediate transition to 5 min of darkness (total test duration of 15 min). All assessments were carried out between 9 am and 7 pm, and treatments randomized to minimize any influence of circadian rhythm. At the end of the experiment, larvae were humanely terminated via anesthetic overdose.

### 2.10 Statistical Analyses

All statistical analyses were carried out using GraphPad Prism (GraphPad Software Inc, San Diego, USA, version 8.0). All data were first tested for normality and homogeneity of variance (Shapiro-Wilks and Bartlett’s Test, respectively) and where parametric test assumptions were met, a one-way ANOVA followed by Dunnett’s multiple comparison test undertaken. Where data were not normal or variances unequal, a Kruskal–Wallis test followed by Dunn’s multiple comparison tests were undertaken. Brain monoamine concentrations were analyzed using a two-way ANOVA followed by Sidak’s post hoc comparison. Data are presented as mean ± SEM, with *p* <0.05 considered the minimal criterion of significance.

## 3 Results and Discussion

This study provides a comprehensive assessment of the bioavailability and neurobehavioral effects of AMI, one of the most widely prescribed tricyclic antidepressants, in zebrafish early life stages, including at environmentally relevant levels. Through a 28 day exposure, we show that AMI bioconcentrates in early life stage zebrafish and induces a pharmacological effect at environmentally relevant concentrations. At supra-environmental concentrations, AMI accelerated the hatch rate and suppressed movement behaviors, but these effects recovered after a 14 day period in AMI free water.

### 3.1 Measured Water Exposure Concentrations

The concentration of AMI measured in the exposure tanks is summarized in Supplementary Table 4. Levels of AMI were stable over the exposure period and ranged from 84 to 105% of the nominal value where measurable. After 14 days of depuration, the mean-measured concentrations were all below 5% of the original dosing nominal.

### 3.2 Mortality, Hatching Rate, and Fish Weight/Length

The rates of mortality and embryo hatching, fish weight, and length are summarized in Supplementary Section 4, Table 6. We found no effect of AMI exposure on growth after 28 days (or after the additional 14 day depuration). This is in contrast with a previous report of reduced fish weight and length in common carp (*Cyprinus carpio*) exposed to 100 μg/L AMI, NOR (and clomipramine) from 8 hpf to 30 dpf.^44^ We would not necessarily expect an impact on growth based on the mechanism of action of AMI, but the absence of such effect here at our higher exposure levels is perhaps surprising given the study findings on common carp. This said, confounding factors can affect fish growth, especially when carrying out assessments in chronic exposure studies. For studies that include early life stages especially, differences in mortality rates and in stocking densities make equitable individual food provision difficult. Moreover, changes in social group dynamics can also have a strong effect on individual fish growth; there were obvious differences between individual fish sizes in our tanks, which may have masked any possible treatment related effect on growth. There was an apparent increase (*p* <0.01) in the % of embryos that had hatched at 72 and 96 hpf, in the 960 nM AMI treatment compared with controls. This finding aligns well with what is reported in the literature.^19,44^ Sehonova *et al.* hypothesized that premature hatching occurs as a result of mitochondrial damage brought about by AMI exposure, and this in turn has the potential to cause hypoxia, a condition previously described to elicit this effect.^44^ Embryos hatching earlier may be more vulnerable if essential developmental milestones are not reached while protected from external elements in the chorion.

### 3.3 Uptake and Transformation of AMI

The concentrations of AMI and NOR measured in the WB, WH, and HNE tissue samples following 28 days of exposure to AMI and after an additional 14 days of depuration are summarized in Supplementary Table 5. Based upon the relatively high lipophilicity of AMI (LogDow at pH 7.4 = 2.96)^45^ and its use as a CNS drug, we expected to see the highest levels of AMI and its metabolite NOR in the brain of our exposed zebrafish, as indicated by measurements of the AMI/NOR level in heads with the eyes removed (HNEs). The BCFs (and pseudo BCFs) we measured in HNE samples for the highest three exposure concentrations (from measured water concentrations) ranged from 44–82 to 3.6–11 L kg^–1^ (summarized in Table 1), respectively, which compare favorably with the only other published study measuring AMI and NOR levels in exposed fish (e.g., BCFs of 50 to 60 for AMI in whole brains of adult gilt-head bream exposed to AMI at 0.2 or 10 μg/L for 7 days).^46^ In the gilt-head bream study, NOR levels were around 10 times lower than AMI, broadly matching our data and indicating that the biotransformation of AMI to NOR in the brain occurs similarly between these two species, even for very different life stages. Interestingly, the ratio between NOR and AMI in the brain was lower at higher water exposure concentrations compared to that for lower exposure levels, which may suggest that the metabolic capacity of the animal had reached saturation at higher exposure levels. David *et al.* reported a BCF of 15 for AMI in the brain tissue of roach (*Rutilus rutilus*) after exposure to treated wastewater effluent containing an AMI concentration of 0.298–0.421 μg/L.^47^ Making comparisons relating to BCFs across these studies is difficult, however, due to a wide range of factors that may affect uptake for the exposure in a wastewater effluent, for example, including differences in the amount of organic matter to which hydrophobic chemicals may absorb, affecting their bioavailability.

**Table 1:** Average Bioconcentration Factors (BCFs) of AMI and Nortriptyline (NOR) in Tissues of Zebrafish Exposed to AMI for 28 Days from Fertilization (2 s.f.)^a^

	average BCF in whole body (L kg^–1^)		average BCF in whole head (L kg^–1^)		average BCF in head with eyes removed (L kg^–1^)	
nominal amitriptyline water concentration (nM)	**AMI**	**NOR**	**AMI**	**NOR**	**AMI**	**NOR**
960	63 ± 7.4	5.4 ± 0.71	180 ± 40	15 ± 3.6	44 ± 5.3	3.6 ± 0.40
96	34 ± 3.5	3 ± 0.27	108 ± 23	7.9 ± 1.4	45 ± 14	3.7 ± 0.85
9.6	62 ± 11	9.7 ± 0.77	247 ± 44	21 ± 3.4	82 ± 27	11 ± 4.1
0.96	105 ± 17	<LOQ	325 ± 81	64 ± 16	<LOQ	<LOQ

a Here, BCFs were calculated from measured water exposure concentrations only (quantified via LC-MSMS as outlined previously). See Supplementary Table S4b for treatment sample sizes as this was variable between both the treatment groups and tissue analyzed. Exposure treatments for which tissue concentrations were below the limit of quantification (<LOQ) are not outlined here.

Notably, the average concentration of AMI and NOR measured in the WH was considerably higher than that measured in the HNE samples, with BCFs (from measured water concentrations) ranging between 108–325 and 7.9–64 L kg^–1^ (as summarized in Table 1), respectively. This indicates a high concentration of AMI is present in the eyes, supporting previous reports that the eyes can serve as a major sink for the accumulation of drugs in fish. In zebrafish larvae, studies on cocaine exposure have found high levels of accumulation in the eyes suggested to be related to drug entrapment in melanophores.^36^ Future studies assessing human drug bioaccumulation in fish tissues might usefully consider the eyes for assessment alongside traditionally sampled tissues such as liver, gonads, and muscle.

Using the whole-body AMI concentration data, we estimate that the measured water concentration at which the human plasma therapeutic concentration (H_T_PC of 0.16–0.96 μM)^27^ would likely be reached in 28 day old zebrafish is between 0.0028 and 0.0168 μM (0.0030–0.0178 μM for nominals). This is considerably higher than any currently reported environmental levels (maximum of 196 ng/L or 0.0006 μM),^5^ suggesting that, under comparable exposure conditions, AMI is unlikely to reach pharmacologically active levels, assuming equivalent species sensitivity. Longer exposure periods (greater than 28 days), however, could result in higher tissue burdens and given that Ziarrusta *et al.* identified 33 metabolites of AMI in gilt-head bream, we may be underestimating the total tissue burden of active metabolites of AMI.^17^

Following 14 days of depuration, over 90% of AMI and NOR was eliminated suggesting that AMI and NOR are not particularly persistent in zebrafish, as is the case in humans (half-lives of 20 and 23–31 h, respectively).^28,29^ To date, no published study has investigated the elimination of AMI in fish following depuration; however, a half-life of 9.4 days has been reported for FLX in Japanese medaka (*Oryzias latipes)* (for a 7 day exposure to 0.64 μg/L^31^), and 9 days in nine-spined stickleback (*Pungitius pungitius*) (for an exposure to 10 μg/L for 14 days^32^), suggesting that FLX is more persistent in fish tissues than AMI.

### 3.4 Brain Monoamine Levels

Measured brain monoamine concentrations are shown in Supplementary Section 5, Table 7a,b. There were no significant changes in whole-brain 5-HT, 5-HIAA, DA, DOPAC, HVA, and NE levels after exposure to AMI for 28 days. Exposure to AMI up to 960 nM also resulted in no changes in the turnover of 5-HT (5-HIAA/5-HT ratio) or DA (DOPAC/DA or HVA/DA ratio) in brains at either 28 or 42 dpf. This is perhaps surprising given that these end points are general characteristics of antidepressant administration in mammals^48,49^ and have been alluded to in fish.^12,50^ One possible reason for the lack of response is that our analyses were undertaken on whole-brain tissues (due to tissue limitations and analytical detection sensitivity), and this may have masked any brain region-specific changes. Melnyk-Lamont *et al.*, for example, found elevated 5-HT, NE, and DA levels only in the midbrain of rainbow trout (*Oncorhynchus mykiss*) exposed to venlafaxine (0.2 and 1 μg/L for 7 days) with no effects observed in the 7 other brain regions assessed.^51^ Interestingly, in our control treatment, dopamine turnover (DOPAC/DA) was higher in zebrafish at 42 days versus 28 days. We do not know the reason for this, but changes in DA levels have been linked with aging in humans.^52^

### 3.5 Expression of 5-HT Transporter/Receptor Genes in the Brain

**Figure 2:**
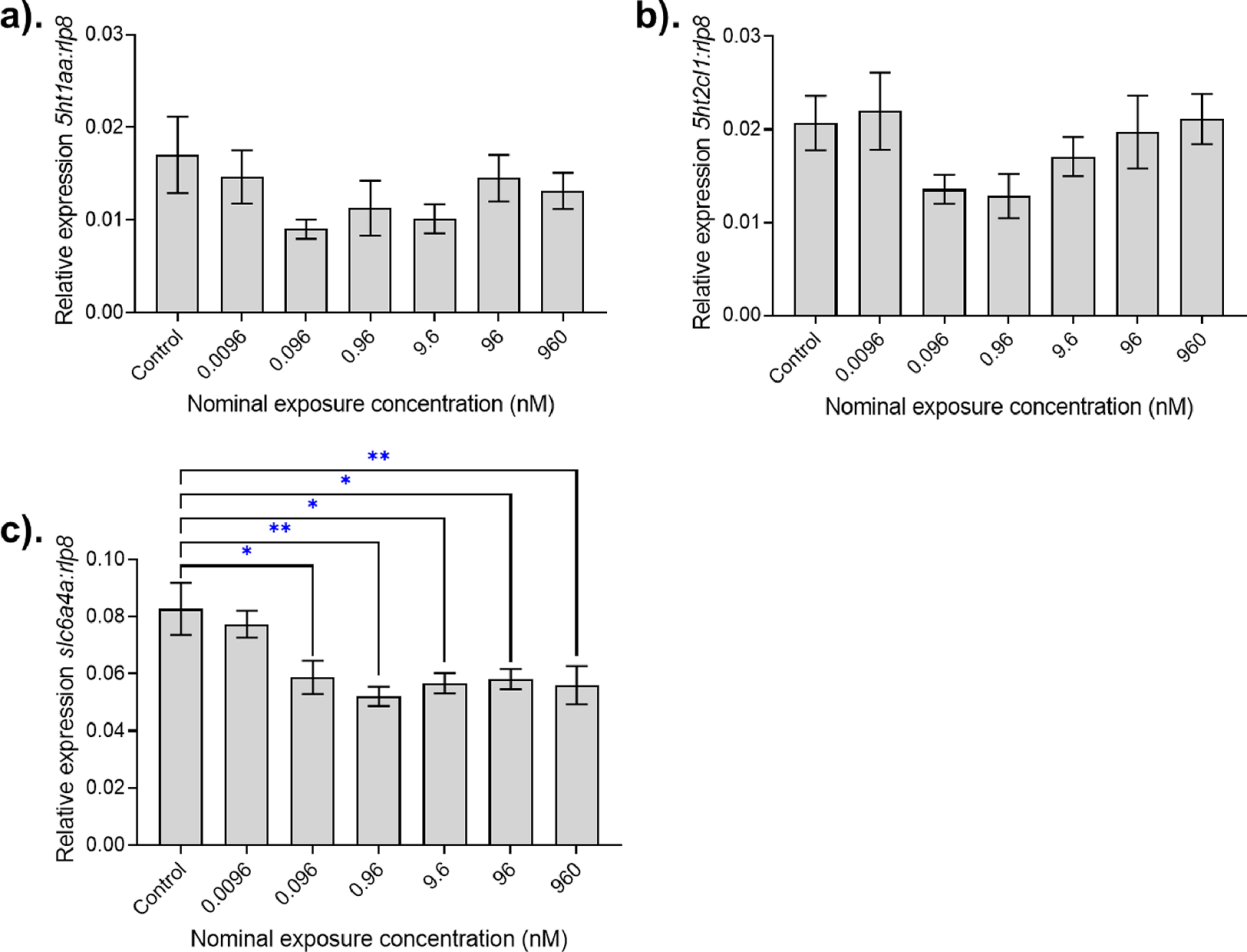
Target gene expression levels in the brains of zebrafish exposed to AMI for 28 days. Relative expressions of the genes (a) *htr1aa*, (b) *htr2cl1*, and (c)*slc6a4a* in the whole heads of 28 dpf zebrafish following a 28 day exposure to amitriptyline are shown. The expression is shown relative to the housekeeping gene *rpl8* (mean ± SEM, *n* = 8 for all treatments with the exception of 0.96 nM, where *n* = 5). Differences in relative expression were assessed using either a Kruskal–Wallis Test (for a and b) or One-way ANOVA (for c) followed by Dunnett’s Test, where significant differences are represented by * (*P* <0.05) and ** (*P* <0.01) versus the control treatment.

The relative expression levels of *htr1a*, *htr2c*, and *slc6a4a* measured are shown in Figure 2. There were no differences in the expression of the housekeeping gene *rpl8* between treatments, the positive control showed no significant intraassay variability, and the NTC samples confirmed that, on each plate, there was no DNA contamination. Quantitative RT-PCR analysis revealed that exposure to AMI for 28 days resulted in significantly lower levels of *slc6a4a* mRNA in animals at an exposure concentration as low as 0.096 nM (Figure 2c). Importantly, the lowest observed effect level occurred for concentrations of AMI that have been detected in the aquatic environment.^53^ This suggests that exposure of fish to AMI at environmentally relevant concentrations can result in pharmacological effects associated with the drug’s primary mechanism of action. These data align with those reported for the SSRI citalopram, in which the expression of *slc6a4a* in whole-brain tissues of adult male zebrafish was reduced, albeit at the higher exposure concentrations of 4, 40, and 100 μg/L (for 2 weeks).^54^ In contrast, Wong *et al.* reported that the expression levels of *slc6a4a* remained unchanged in whole-brain tissue of adult male zebrafish following a 2-week exposure to 100 μg/L FLX^15^ perhaps reflecting the differences in compound potency, life stage, or exposure period between the two studies.

Exposure to AMI for 28 days did not, however, result in altered brain mRNA levels of *htr1aa* or *htr2c*. This is perhaps not especially surprising, given that 5-HT receptors are not the primary target for AMI, albeit *htr1aa* has previously been shown to be downregulated in male adult zebrafish exposed to 5 mg L^–1^ FLX (in dominant but not subordinate males) suggesting brain levels can be affected.^55^ Other reports of altered serotonin receptor gene expression in whole-body homogenates have reported that, for exposure to FLX, the expression of *htr1aa* in whole zebrafish was inhibited in a dose-dependent manner,^23^ and *htr2c* mRNA levels were downregulated in zebrafish embryo-larvae at concentrations including those of environmental relevance.^56^ As is the case with brain monoaminergic concentrations, expression of target gene expression levels will vary across different brain regions,^20,57^ and as such, the sampling of whole heads here likely do not provide sufficient resolution to detect small changes in the regional levels of some genes.

### 3.6 Fish Behavior

#### 3.6.1 General Locomotor Activity

**Figure 3:**
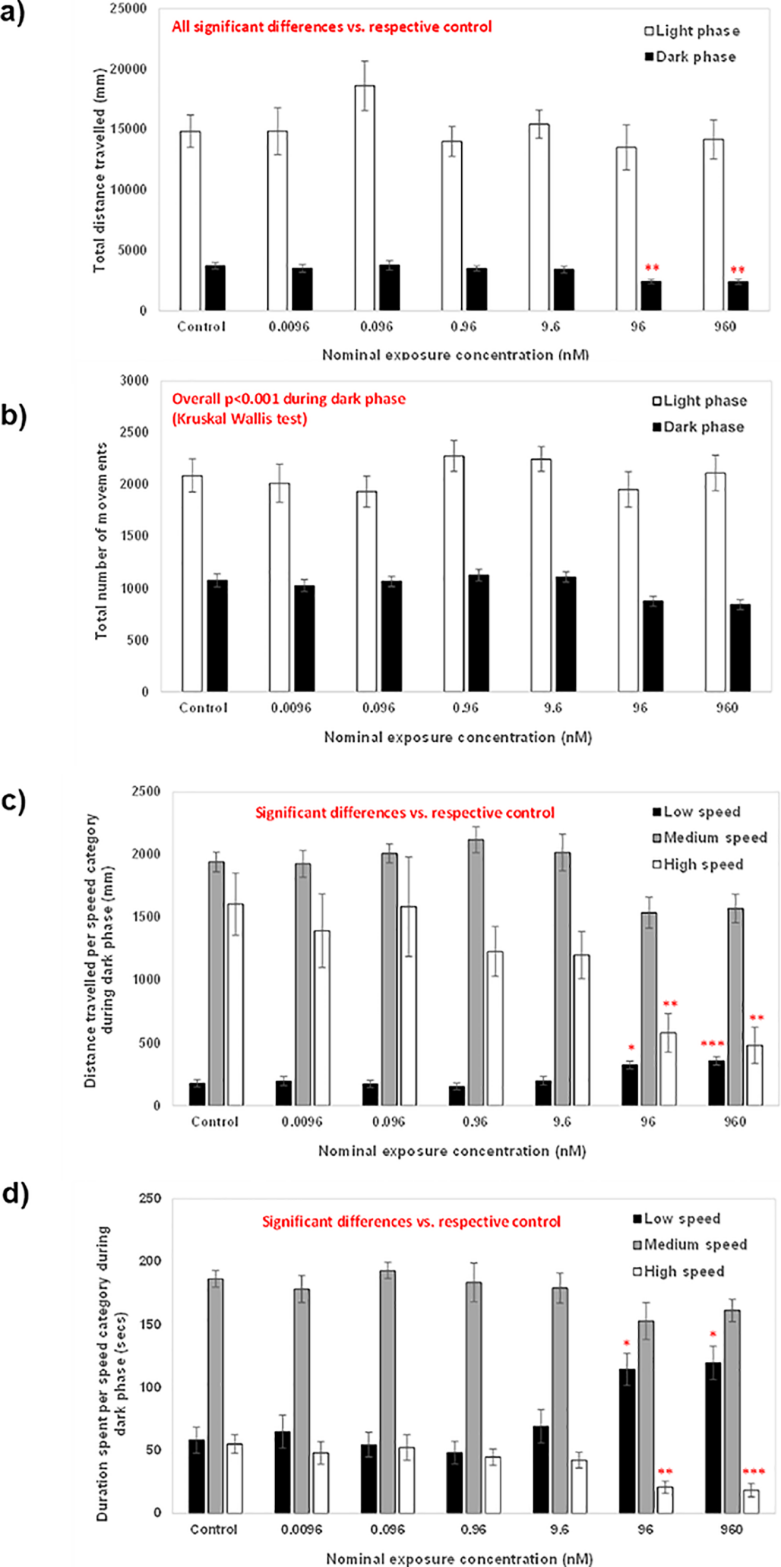
Summary of the results of the behavioral assessment of zebrafish exposed to AMI for 28 days. Movement parameters of 28 dpf fish across the entire test arena during the 15 min assessment period, including a 10 min period of light and 5 min period of dark. (a) Total distance traveled during the light (shaded white) and dark (shaded black) phases, (b) total number of movements during the light (shaded white) and dark (shaded black) phases, (c) distance traveled at low (shaded black), medium (shaded grey), and high (shaded white) speeds, and (d) duration spent traveling at low (shaded black), medium (shaded grey), and high (shaded white) speeds (mean ± SEM, *n* = 16). The speed of movements were categorized as follows, whereby low represents <5 mm/s, medium represents 5–20 mm/s, and high represents >20 mm/s. These were designed simply to show broad differences in this aspect of movement. Differences between AMI-exposed fish versus those in the control treatment were assessed using Kruskal–Wallis and Dunn’s test. Significant differences are represented by * (*P* <0.05), ** (*P* <0.01), and *** (*P* <0.001).

Behavioral assessment of AMI-exposed zebrafish revealed a concentration-dependent reduction in distance traveled, number of movements, time spent moving, and speed of movement, but only during periods of darkness (results are summarized in Figure 3). Fish were found to exhibit significantly reduced locomotor activity at the highest two exposure concentrations (96 and 960 nM), which aligns with several previous studies.^12,16,50,58−60^ Sehonova *et al.*, for example, reported that zebrafish exposed to 300 μg/L AMI (equivalent to the 960 nM concentration in the current study) from <16 cell stage to 144 hpf exhibited significantly reduced swimming distances during periods of darkness but not during light.^59^ Sedation is a known side effect of TCA antidepressants and is likely related to antihistaminergic activity.^61^ This is possible here given the genes encoding the three known zebrafish histamine receptor orthologues, *hrh1, hrh2*, and *hrh3*, are expressed from as early as 5 dpf, and treatment with agonists is known to result in reduced swimming activity.^62,63^ It should be noted, however, that, after the 14 day depuration period, no differences in any measures of general locomotor activity were detected (data not shown).

#### 3.6.2 Thigmotaxis

In contrast with the clear effect of AMI exposure on general locomotor activity, no significant alteration of thigmotaxis was observed at any treatment level or between exposure and depuration periods. This is despite clearly observed thigmotaxis, particularly during the light phase when (presumably) juvenile fish may feel more at risk of predation (Supplementary Section 7, Figure 5). This also contrasts with some previous reports of antidepressant induced anxiolysis in zebrafish (e.g., ref (64)). Although we quantified thigmotaxis only in the horizontal plane, we did anecdotally observe fish exposed to the higher concentrations preferentially located in the upper sections of the tanks. It may, therefore, be the case that the use of a three-dimensional place preference test would be more revealing. Indeed, Demin *et al.* and Meshalkina *et al.* reported anxiolytic effects of AMI in zebrafish using the novel tank diving test.^12,16^ In tests with fish, behavioral effects have been detected at lower tested concentrations when individuals have been subjected to an additional stressor (e.g. refs (15) and (65)). Thus, the application of an additional stressor (and one that is stronger than light transition) to any future assessments of the impact of exposure to antidepressants in fish may provide an additional layer of sensitivity to the measured end points. Moreover, the addition of such stimuli may better simulate conditions in the natural environment where fish will be exposed to multiple stressors spanning predation threat, limitations in food supply, or competition for mates. In the current study, all AMI exposure-induced behavioral phenotypes (reduced distance travelled and speed of movement) were found to recover following the 14 day period of depuration, further supporting that these effects were treatment related. In addition, compared with previous data, this also highlights the relative lack of persistence of AMI in fish tissues compared with other antidepressants such as FLX.^14^

Overall, our data show that exposure of zebrafish from 0 to 28 dpf affects the development, physiology, and behavior of zebrafish, with the lowest observed effect concentrations being of environmental relevance. The lowest observed effect concentration in this study was 0.096 nM, at which the mRNA levels of the 5-HT transporter *slc6a4a* were significantly lower than controls, and a water exposure concentration well within the range of measured AMI environmental concentrations. The behavioral end points we measured, however, were only affected at AMI concentrations several orders of magnitude above those measured in surface waters questioning the likelihood that current environmental concentrations of AMI are, by themselves, sufficient to induce any harmful impacts for fish in the natural environment. It should be recognised that a limitation in our behavioral assessments is the possible influence of circadian rhythms that are receiving increasing attention for studies on pollution impacts on behaviors.^66^ We conducted the studies on behavior between 9 am and 7 pm, and although experimental conditions were randomized to minimize the influence of circadian rhythms, any potential impact of timing remains untested and unaccounted for in the statistical analysis. This, in turn, may mask any potential influence of circadian rhythms that could have a bearing on the observed lack of behavioral response and implied low risk associated with exposure to this drug. Also, when considering the environmental risk posed by the range of widely used antidepressants, the mechanisms of action of many of these drugs are similar which may indicate a high likelihood for additive effects, and the targets on which they act show strong molecular and functional conservation across diverse taxonomic groups.^4^ This may mean the environmental risk is higher for mixtures of antidepressant (and some other neuroactive) compounds than for other drug classes that do not share the same molecular mechanisms of action. Greater focus on environmentally realistic mixtures of similarly acting neuroactive drugs is thus warranted to fully understand the risk that these contaminants may pose to fish in the wild. Nevertheless, our data suggest that exposure of zebrafish to AMI in isolation at environmentally detected levels is unlikely to significantly impair the locomotor activity of juvenile zebrafish, exert anxiolytic effects, or alter monoamine brain chemistry.

## Supporting Information Available

Photographs of the experimental setup; detailed descriptions of the water exposure parameters (flow rates and water quality), and methods used and measurements of amitriptyline in the water (during the saturation, exposure, and depuration periods); rationale, methods, and results of the feeding trial; measurements of amitriptyline in tissue samples; methods used and effects of amitriptyline on apical end points including mortality, hatch rate, and growth; brain monoamine analysis (HPLC) methods and results; 5-HT transporter/receptor gene analysis methods; and behavioral analysis with a figure illustrating zonal preferences in the test arena (PDF)
