# Supplementary material for: Exposure Effects of Environmentally Relevant Concentrations of the Tricyclic Antidepressant Amitriptyline in Early Life Stage Zebrafish
Source: Environ Sci Technol. 2024 Jul 17;58(30):13194–204. doi: 10.1021/acs.est.3c08126 (PMC11295126; doi:10.1021/acs.est.3c08126)
Supplement: Supplementary file 1 — es3c08126_si_003.pdf [file es3c08126_si_003.pdf]

Title Page: Supporting Information

Exposure effects of environmentally-relevant concentrations of the tricyclic antidepressant amitriptyline in early life stage zebrafish.

*Sophie L. Gould[a], Matthew J. Winter[a], Maciej Trznadel[a], Anke Lange[a], Charles M. Hamilton[a], Rebekah J. Boreham[a] Malcolm J. Hetheridge[a], Andrew Young[b], William H. J. Norton[b] and Charles R. Tyler[a]**

[a]Biosciences, Faculty of Health and Life Sciences, University of Exeter, Stocker Road, Exeter, Devon, EX4 4QD, UK.

[b]Department of Genetics and Genome Biology, College of Life Sciences, University of Leicester, University Rd, Leicester, LE1 7RH, UK.

Corresponding author

* Charles R. Tyler, Biosciences, Faculty of Health and Life Sciences, University of Exeter, Exeter, Devon, EX4 4QD, UK.

Email: C.R.Tyler@exeter.ac.uk

**Summary: Supporting Information**	
Number of pages	22
Number of figures	5
Number of tables	8

Supporting Information

Section 1: Water exposure parameters

Experimental set up

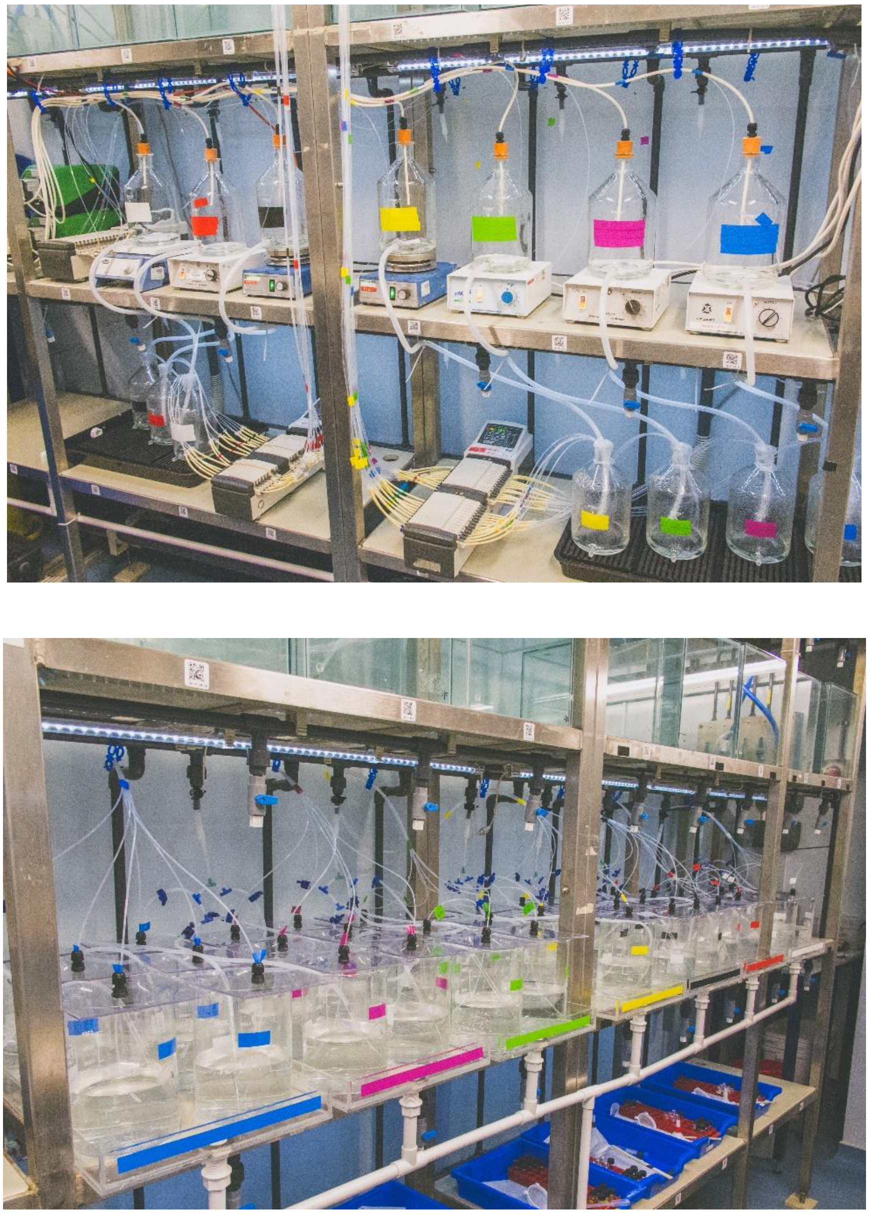

Supp Figure 1. *Photographs of the flow through system set up: (a) the mixing chambers and peristaltic pumps, and (b) the test vessels, where each colour represents a different amitriptyline exposure concentration.*

Flow rates

Supp Table 1. *Average flow rate of the amitriptyline stock solution and RO water over the 28-day exposure period (mean ± SEM, n = 5).*

Concentration (nM)	Amitriptyline stock flow rate (ml/min)	RO water flow rate (ml/min)
**960**	0.46 ± 0.0006	18.81 ± 0.0914
**96**	0.46 ± 0.0015	18.44 ± 0.1475
**9.6**	0.46 ± 0.0009	18.00 ± 0
**0.96**	0.42 ± 0.0015	18.25 ± 0.0945
**0.096**	0.43 ± 0.0014	18.00 ± 0
**0.0096**	0.46 ± 0.0007	20.00 ± 0
**Control**	0.47 ± 0.0123	19.12 ± 0.2266

Water quality parameters

Water quality parameters, including; pH, temperature, dissolved oxygen (FireStingO_2 fiber-optic oxygen meter, PryoScience Sensor Technology) and ammonia (Tropic Marin Ammonia/Ammonium Test Kit) were measured throughout the experiment on a weekly basis (pH was measured twice a week).

Supp Table 2. *Tank water quality parameters, including pH, temperature and dissolved oxygen. Outlined as the mean (SEM) over the entire 42-day duration of the exposure where n = 5.*

Concentration (nM)	pH	Temperature (°C)	Dissolved oxygen (%)	Ammonia (mg L^{-1})
**960**	6.90 ± 0.02	27.61 ± 0.05	89.36 ± 0.72	0.48 ± 0.08
**96**	6.91 ± 0.04	27.53 ± 0.05	86.95 ± 0.96	0.54 ± 0.10
**9.6**	6.90 ± 0.02	27.57 ± 0.05	86.59 ± 0.86	0.37 ± 0.03
**0.96**	6.95 ± 0.06	27.64 ± 0.05	88.47 ± 0.87	0.43 ± 0.07
**0.096**	6.89 ± 0.02	27.62 ± 0.05	88.09 ± 0.83	0.54 ± 0.10
**0.0096**	6.90 ± 0.02	27.51 ± 0.04	89.14 ± 0.82	0.44 ± 0.11
**Control**	6.84 ± 0.02	27.52 ± 0.06	90.39 ± 0.68	0.45 ± 0.08

Measurement of AMI in water samples

For analysis, 800 µl of tank solution, plus 200 µl of AMI-d_3 hydrochloride solution (CAS: 342611-00-1) that had been diluted to 96 nM in methanol, was transferred to a small glass vial prior to vortexing. Once well mixed, 100 µl was transferred to a deep 96 well plate for analysis by LC-MS/MS. Analyses of water samples were performed using a TSQ Vantage triple quadrupole mass spectrometer. The mass spectrometer was equipped with a heated electrospray (HESI II) source (ThermoFisher Scientific, Hemel Hempstead, UK). The HESI probe was operating in positive mode; an ion-spray voltage of 4.0 kV, heated capillary temperature was set at 270 °C and the vaporizer temperature was 350 °C. Nitrogen was employed as a sheath and auxiliary gas at a pressure of 60 and 2 arbitrary units, respectively. The argon CID gas was used at a pressure of 1.5 mTorr and the optimum CE for each transition was selected. Quantification was performed by monitoring two characteristic MRM transitions for AMI and one for the d_3- AMI internal standard. Chromatographic separation was achieved using a reversed-phase, 3 µm particle size, C18 Hypersil GOLD column (50 mm × 2.1 mm id., Thermo Scientific, San Jose CA, USA). All analytes were separated using a linear gradient with a flow rate of 500 µL min^{-1}. The autosampler temperature was maintained at 8˚C, while the column was kept at room temperature.

Saturation period concentrations

Supp Table 3. *Measured concentrations of amitriptyline in exposure water during the saturation phase (mean ± SEM, n = 5). Note only the highest four concentrations of AMI in water were above the LOQ of the LC-MS/MS method used.*

**Nominal exposure concentration (nM)**	**Mean measured concentration ± SEM (nM)**	**Measured concentration as % of nominal ± SEM**
960	837 ± 9.47	87.20 ± 0.98
96	86 ± 1.01	89.87 ± 1.06
9.6	9.3 ± 0.10	97.38 ± 1.06
0.96	0.81 ± 0.01	84.24 ± 1.35
0.096	<LOQ	<LOQ
0.0096	<LOQ	<LOQ
Control	<LOQ	<LOQ

Exposure and depuration period concentrations

Supp Table 4. Results of the chemical analysis of water samples from the 28-day exposure and 14 day depuration of zebrafish to AMI (2 s.f.). *Measured concentrations of amitriptyline in exposure water across the duration of the 42-day experiment expressed as the % of the nominal (mean ± SEM, n = 5).*

Nominal concentration (nM)	Measured concentration as % of nominal						
	Day 0	Day 7	Day 14	Day 21	Day 28	Day 35	Day 42
	Exposure					Depuration	
960	84 ± 0.74	87 ± 0.66	98 ± 0.79	95 ± 0.44	101 ± 2.1	2.7 ± 0.02	1.5 ± 0.05
96	83 ± 1.1	94 ± 0.69	95 ± 1.3	92 ± 0.43	95 ± 1.6	3.7 ± 0.08	2.2 ± 0.03
9.6	90 ± 0.64	91 ± 1.4	101 ± 2.2	95 ± 1.6	101 ± 2.7	4.9 ± 0.17	2.9 ± 0.13
0.96	72 ± 1.1	82 ± 2.7	85 ± 3.3	87 ± 1.8	92 ± 2.5	<LOQ	<LOQ
0.096	<LOQ	<LOQ	<LOQ	<LOQ	<LOQ	<LOQ	<LOQ
0.0096	<LOQ	<LOQ	<LOQ	<LOQ	<LOQ	<LOQ	<LOQ
Control	<LOQ	<LOQ	<LOQ	<LOQ	<LOQ	<LOQ	<LOQ

Section 2: Feeding trial

Rationale and aim

To avoid dilution of the exposure solution that would come with administering *Artemia nauplii* and minimise any contribution of trophic transfer from live prey and biomagnification of amitriptyline in tissues, a dry food ONLY diet was used. Furthermore, this allowed control of the mass of feed in each tank relative to the number of surviving individuals i.e. this allowed adjustment of the mass of dry food to account for mortalities. First, the growth of healthy zebrafish larvae was established from 5 to 42 dpf (duration of the experiment where fish were to be fed exogenously) when fed an optimum standard aquarium diet. Using this information, 3 feeding regimes were tested to determine the optimum feeding protocol with dry feed only.

Zebrafish weight measurements from 5 to 42 dpf

Zebrafish were housed in accordance to the standard aquarium guidelines as follows: from 5-7 dpf, larvae were housed in 1 L static tank and fed 1 dry feed (1/2 scoop <100 ZEBRAFEED by Sparos) and 3 rotifer feeds (3ml) daily. From 7 to 20 dpf, larvae were housed in 3.5 L tanks with a drip rate of 1-2 drips per second and fed 1 dry feed (1 scoop <100 ZEBRAFEED by Sparos) and 3 rotifer feeds (5 ml) daily. From 21-30 dpf, larvae were housed in 3.5 L tanks with a drip rate of 1-2 drips per second and fed 2 dry feeds (1/2 scoop 100-200 ZEBRAFEED by Sparos), 1 rotifer feed (5 ml) and 1 artemia feed (1 ml) daily. From 31-40 dpf, larvae were housed in 3.5 L tanks with a drip rate of 2-3 drips per second and fed 2 dry feeds (1 scoop 100-200 ZEBRAFEED by Sparos) and 1 artemia feed (2 ml) daily. From 41 dpf onwards, larvae were housed in 8 L tanks with a drip rate of 2-3 drips per second and fed 2 dry feeds

(2 scoops 200-400 ZEBRAFEED by Sparos) and 1 artemia feed (5 ml) daily. At 5-day intervals commencing from 5 to 45 dpf, 10 fish were removed from stock tanks (2/3 from each tank) and placed in a plastic Petri dish containing 50ml reverse osmosis (RO) water. Following schedule 1 termination, fish length (measured using Leica M205C with a Leica DMC4500 camera using 1x panio objective and LAS X programme) and weight were measured (Figure S2). At 5 and 10 dpf, to increase measurement accuracy due to their small size, body weight measurements were made by pooling two fish and then dividing this measurement by 2 to provide an average individual weight for those two individual fish. From 15dpf onwards, individual fish weights were taken.

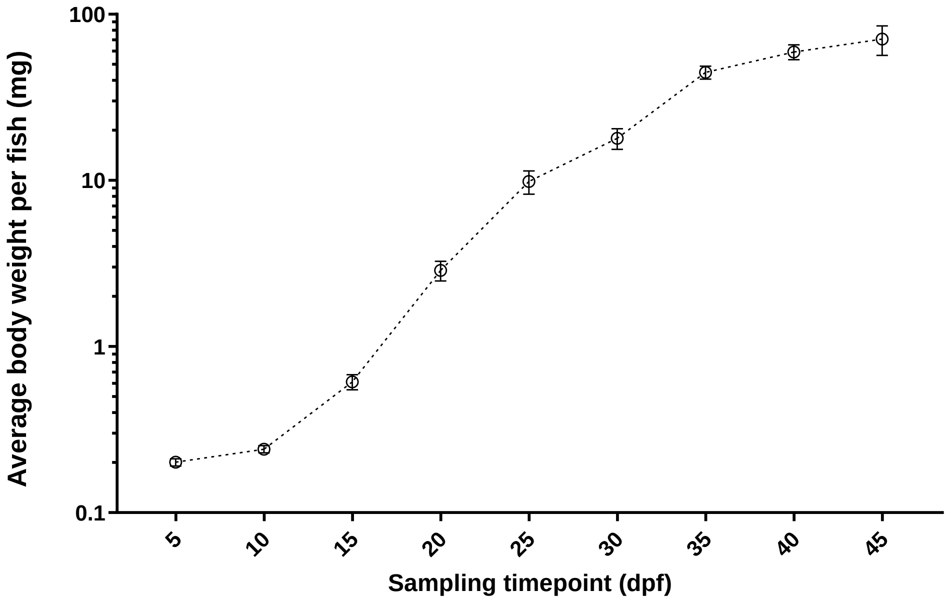

Supp Figure 2. *Zebrafish larva growth over time as depicted by weight, when fed standard aquarium diet of Artemia nauplii, rotifers and dry food combined (mean ± SEM, N = 5 at 5 and 10dpf and 10 from 15dpf onwards). The y-axis is presented on a logarithmic scale.*

Feeding trial

Adult fish were spawned and embryos collected as outlined in section 2.3. Embryos-larvae were kept in Petri Dishes containing 50 ml aquarium water (75% daily water change) at 28 ± 1°C until 5 dpf. From the onset of independent feeding (5 dpf), larvae were fed Zebrafeed, Sparos dry food at one of three quantities, 50, 175 or 300% of total body weight (as calculated above), or the standard aquarium feed (outlined above). The daily body weights of larvae were selected from Figure S2. Each treatment had 2 replicate tanks, each housing 55 larvae at 5 dpf and then until 20 dpf, at 5-day intervals, fish were removed from each tank for length and weight measurements. At 5 dpf, to increase measurement accuracy due to their small size, body weight measurements were made by pooling two fish and then dividing this measurement by 2 to provide an average individual weight for those two individual fish (2 fish sampled per tank). From 10dpf onwards, individual fish weights were taken (10 fish sampled per tank).

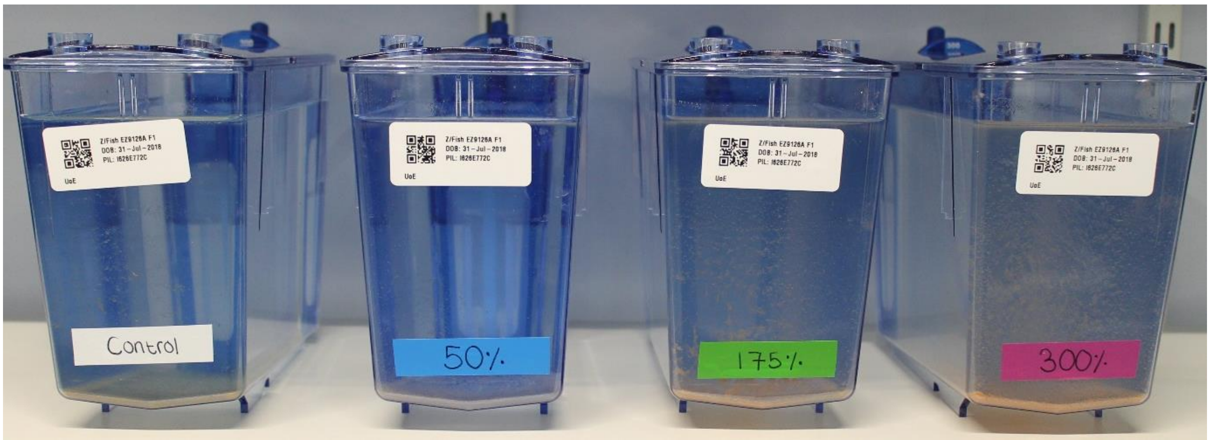

Supp Figure 3. *Build-up of dry feed in tanks at 20 dpf: standard aquarium diet (white), 50% (blue), 175% (green) and 300% (pink) of body weight.*

As seen from Figure S3, food build up in the 175 and 300% tanks was excessive, particularly in the 300% tanks which is likely why at 20 dpf, larvae in one of the 300% tanks died (ammonia/bacteria accumulation) due to excess debris. Furthermore, it was observed that larvae in the 50% tank spent the majority of their time swimming at the water's surface, indicative of searching/waiting for food. It should be noted that tanks were not cleaned over the 15-day period. Larvae fed on the control diet (standard aquarium diet) had the highest growth rate which was expected as this

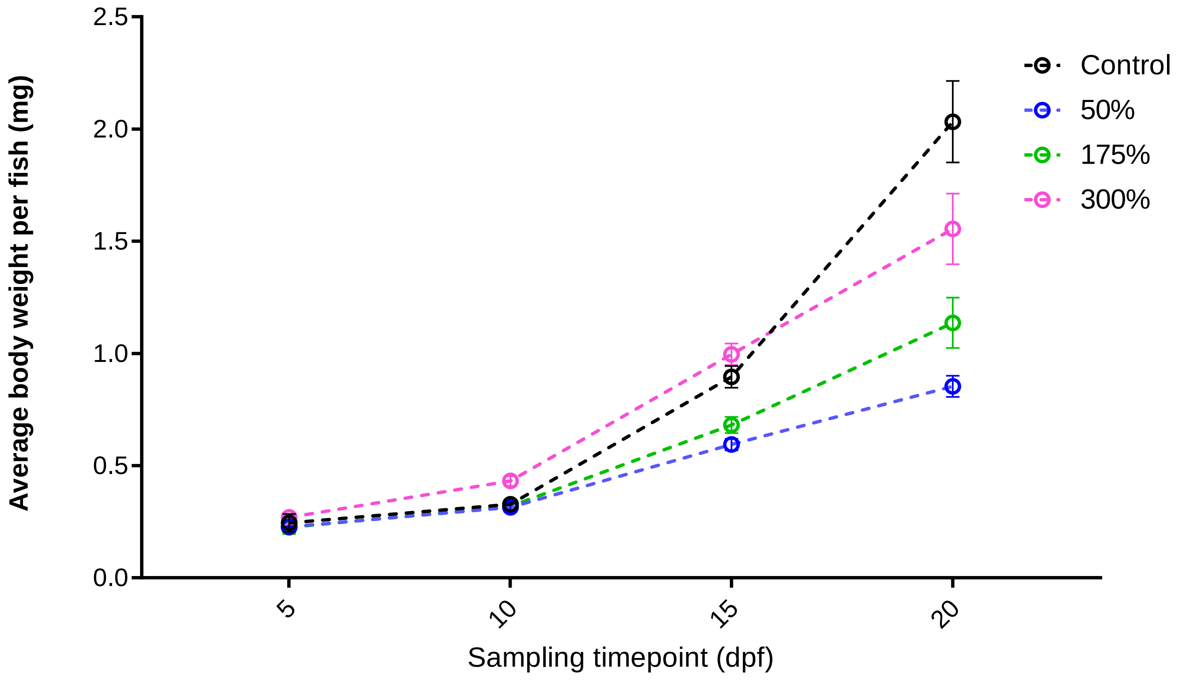

Supp Figure 4. *Zebrafish larvae growth over time when fed different percentages of their body weight in dry food compared to the standard aquarium control diet (mean ± SEM, N = 2 at 5 dpf and 10 from 10 dpf onwards).*

feeding regime has been established to be optimum for growth in this aquarium environment (Figure S4). All three dry feed only regimes resulted in larvae having reduced growth compared to the control (Figure S4). The cleanliness of the tanks diminished (build-up of debris) as the amount of food added increased, and is

ultimately what led to unfavourable living conditions, particularly for those on the 300% diet. However, the 300% treatment yielded body weights most similar to larvae fed on the standard aquarium diet so to enable sufficient growth, for the main exposure study, larvae were fed 300% of their body weight in dry feed from 5 dpf and tanks were cleaned daily. When it was clear this ration was too great (i.e. food continued to accumulate in the tanks as fish were not eating it all), the % body weight of food given was adjusted accordingly.

Section 3: Measurement of AMI in tissue samples

Once defrosted, individual samples (WB, WH and HNE) were transferred to a new 2 ml Eppendorf containing 600 µl of acetonitrile:water 1:1 mix with 25 µg L^{-1} internal standards of AMI and NOR spiking solution, alongside a stainless steel ball bearing. Samples were then homogenised in a Tissuelyser (Qiagen) at 300 Hz for 3 minutes before being centrifuged at 160,000g for 1 hour at room temperature. 250 µl of the supernatant was then transferred to HPLC glass vials lined with inserts for LCMS/MS analysis. At the highest treatment level, AMI concentration was measured in head samples (with and without the eyes), alongside the remaining trunk for the same animal. Heads were analysed singularly for the higher concentrations but were pooled in groups of 3 for the lower concentrations.

The analysis of AMI and NOR was again undertaken on a TSQ Vantage triple quadrupole mass spectrometer as detailed above. All conditions were the same as for the water samples except that the analytes were separated using a linear gradient of water and methanol, both containing 0.1% formic acid. The initial conditions for the gradient consisted of 20% methanol with formic acid, which was increased to 100% in 1.5 min and maintained for another 1.5 min before returning to the initial 5% (flow rate

was again 500 µL min^{-1}). In addition, the autosampler temperature was set at 6˚C in this case. To assess analyte recoveries, two WB tissues from the 28 dpf-sampled control group were processed as above, but after homogenization spiked with a known amount of AMI and NOR and left for 30 minutes to allow for matrix binding. The samples were then processed as before. Analyte recoveries were found to be 90 ± 1% for AMI and 103 ± 7% for NOR. It should be noted that for some of the lower concentrations, tissues were pooled as levels were too low for analysis on individual animals.

Supp Table 5. Results of the chemical analysis tissue samples from the 28-day exposure and 14 day depuration of zebrafish to AMI (2 s.f.). Average tissue concentrations of amitriptyline and nortriptyline in the whole-body tissues, whole head tissues and head tissues with the eyes removed, of zebrafish exposed to amitriptyline, at a range of concentrations, for 28 days and then following a 14-day period of depuration in clean water up to 42 dpf (mean ± SEM, n = see table).

Nominal exposure concentration (nM)	Concentration in whole body (mg kg^{-1})			Concentration in whole head (mg kg^{-1})			Concentration in head with eyes removed (mg kg^{-1})		
	Amitriptyline	Nortriptyline	Sample size	Amitriptyline	Nortriptyline	Sample size	Amitriptyline	Nortriptyline	Sample size
**28dpf**									
960	16 ± 1.8	1.28 ± 0.17	10	45 ± 10	3.8 ± 0.90	7	11 ± 1.3	0.89 ± 0.010	8
96	0.85 ± 0.084	0.069 ± 0.006	10	2.7 ± 0.56	0.19 ± 0.033	4	1.1 ± 0.35	0.089 ± 0.021	4
9.6	0.15 ± 0.029	0.024 ± 0.002	10	0.63 ± 0.11	0.054 ± 0.009	3	0.21 ± 0.071	0.028 ± 0.012	4
0.96	0.023 ± 0.004	<LOQ	9	0.072 ± 0.018	0.014 ± 0.004	3	<LOQ	<LOQ	4
**42dpf**									
960	0.28 ± 0.13	0.036 ± 0.014	10	0.49 ± 0.094	0.047 ± 0.011	7	0.13 ± 0.035	0.021 ± 0.003	7
96	0.032 ± 0.004	<LOQ	10	0.088 ± 0.011	0.012 ± 0.002	3	0.026 ± 0.005	<LOQ	3
9.6	0.010 ± 0.003	<LOQ	10	<LOQ	<LOQ	3	<LOQ	<LOQ	3
0.96	<LOQ	<LOQ	9	<LOQ	<LOQ	3	<LOQ	<LOQ	3

Section 4: Assessment of apical endpoints

Supp Table 6. *Effects of amitriptyline exposure on zebrafish apical endpoints. (a) mortality, represented as a cumulative measure at 28 dpf, (b) hatching rate, which was recorded at 24-hour intervals until 120 hpf, (c) body weight and (d) body length (mean ± SEM, n = 5 for (a) and (b) and N = 38-60 for (c) and (d)). Differences in endpoints between drug-exposed fish and the controls were assessed using Kruskal-Wallis and Dunn's Multiple Comparison tests, where significant differences are represented by ** (P<0.01).*

**Concentration (nM)**	**(a). Mortality (%)**	**(b). Hatch rate (%)**				**(c). Body weight (mg)**		**(d). Body length (mm)**	
		**48hpf**	**72hpf**	**96hpf**	**120hpf**	**28dpf**	**42dpf**	**28dpf**	**42dpf**
960	47.78 ± 6.79	1.72 ± 0.87	43.44 ± 10.07	92.38 ± 2.93 **	100 ± 0.00	4.21 ± 0.59	13.12 ± 1.58	7.43 ± 0.23	9.72 ± 0.32
96	20.83 ± 5.69	0.00 ± 0.00	9.10 ± 2.16	77.48 ± 4.38 **	99.78 ± 0.25	4.24 ± 0.40	13.97 ± 1.42	7.81 ± 0.21	9.98 ± 0.32
9.6	18.22 ± 3.72	0.00 ± 0.00	18.08 ± 5.83	82.41 ± 2.43	100 ± 0.00	3.48 ± 0.33	11.99 ± 1.27	7.53 ± 0.19	9.62 ± 0.32
0.96	20.22 ± 3.41	0.00 ± 0.00	10.42 ± 1.95	77.34 ± 3.14	99.53 ± 0.29	2.98 ± 0.44	10.79 ± 1.31	7.24 ± 0.23	9.42 ± 0.31
0.096	16.22 ± 4.77	0.00 ± 0.00	6.27 ± 1.44	76.92 ± 3.40	100 ± 0.00	4.75 ± 0.41	12.23 ± 1.43	8.09 ± 0.22	9.57 ± 0.32
0.0096	20.00 ± 3.90	0.00 ± 0.00	15.18 ± 3.38	80.75 ± 3.17	99.73 ± 0.27	4.38 ± 0.48	9.98 ± 1.19	7.89 ± 0.23	9.46 ± 0.28
Control	24.44 ± 3.72	0.23 ± 0.23	7.83 ± 4.02	67.99 ± 5.54	99.77 ± 0.23	3.63 ± 0.34	11.86 ± 1.52	7.46 ± 0.19	9.57 ± 0.35

Section 5: Brain Monoamine Analysis via HPLC

At 28 and 42dpf, a subset of fish from each of the control, 0.096nM and 960nM treatments (representing pharmacologically and environmentally relevant concentrations respectively) were terminated and their brains removed in E3 medium (see recipe below) under a dissection microscope. Samples were then snap frozen in Eppendorfs in liquid nitrogen and stored at -80°C until further processing. For analysis of monoamine levels, brains were weighed, 100 µl of 0.1 N perchloric acid was added, and then they were homogenised using a micro-pestle. Samples were then centrifuged at 4°C, 12000 x g for 15 minutes, before the supernatant was collected. HPLC analysis of the selected neurotransmitter supernatants was carried out using the method of Carreno Gutierrez et al. (2018), on an ANTEC-HPLC system with electrochemical detection [1]. The mobile phase consisted of 75 mM sodium dihydrogen phosphate, 1 mM EDTA and 0.6 mM octane sulphonic acid (OSA) in deionised water containing 10% methanol. Samples were quantified based upon a one-point calibration using a 50 nM standard of each of the neurotransmitters being analysed. Neurotransmitters measured were: 5-HT, 5-hydroxyindoleacetic acid (5-HIAA, the main metabolite of 5-HT), norepinephrine (NE), dopamine (DA), 3,4-Dihydroxyphenylacetic acid (DOPAC, the major metabolite of DA) and homovanillic acid (HVA, the product of degraded DA). The ratios of 5-HIAA/5-HT, DOPAC/DA and HVA/DA were calculated to assess monoamine turnover in the brain.

E3 Medium recipe, as described in Cold Spring Harbor Protocols (2011).

- 34.8 g NaCl
- 1.6 g KCl
- 5.8 g $CaCl_2 \cdot 2H_2O$
- 9.78 g $MgCl_2 \cdot 6H_2O$

Supp Table 7. *Neurochemical analysis of zebrafish whole brain tissues following a 28-day amitriptyline exposure and 14-day depuration period in clean water. Here, (a) the concentrations of single monoamines including 5-HT, 5-HIAA, DA, DOPAC, HVA and NE were measured, alongside (b) the ratios of 5-HIAA/5-HT, DOPAC/DA and HVA/DA are shown, reflecting 5-HT and DA turnover, respectively (mean ± SEM, n = 7/8 per treatment). Differences in neurotransmitter concentrations (nM) were assessed using a two-way ANOVA followed by Sidak's post hoc comparison, where significant differences are represented by ** (P<0.01).*

**(a).**	**Singular monoamine analysis**											
**Concentration (nM)**	**5-HT**		**5-HIAA**		**DA**		**DOPAC**		**HVA**		**NE**	
	**28 dpf**	**42 dpf**	**28 dpf**	**42 dpf**	**28 dpf**	**42 dpf**	**28 dpf**	**42 dpf**	**28 dpf**	**42 dpf**	**28 dpf**	**42 dpf**
**960**	1092.09 ± 80.45	1302.91 ± 219.29	832.32 ± 141.30	2137.82 ± 1263.00	832.32 ± 141.3	2137.40 ± 1263.00	6072.1 ± 844.10	5574.90 ± 459.95	629.85 ± 128.37	8352.40 ± 5250.30	17986.60 ± 3557.13	12182.43 ± 3316.57
**0.096**	18155.35 ± 12046.69	1446.13 ± 225.02	1448.13 ± 280.10	2672.82 ± 1790.53	1448.1o ± 280.10	2672.80 ± 1790.50	7973.10 ± 2012.10	6305.70 ± 515.52	6098.20 ± 4748.80	1260.20 ± 383.00	39993.69 ± 19984.03	11351.18 ± 1788.87
**Control**	1555.99 ± 193.41	1418.11 ± 261.06	1444.88 ± 382.48	651.23 ± 180.02	1444.90 ± 382.48	651.23 ± 180.02	6201.10 ± 564.57	7090.30 ± 767.69	630.57 ± 184.08	13281.00 ± 12129.00	15848.56 ± 3936.09	29180.32 ± 19604.44

**(b).**	**Monoamine turnover analysis**					
**Concentration (nM)**	**5-HIAA/5-HT**		**DOPAC/DA**		**HVA/DA**	
	**28 dpf**	**42 dpf**	**28 dpf**	**42 dpf**	**28 dpf**	**42 dpf**
**960**	0.76 ± 0.14	1.28 ± 0.63	2.71 ± 1.36	5.38 ± 0.99	0.26 ± 0.08	8.08 ± 5.87
**0.096**	0.46 ± 0.13	1.78 ± 1.11	3.21 ± 0.88	5.64 ± 1.36	5.66 ± 4.93	0.91 ± 0.27
**Control**	1.03 ± 0.33	0.48 ± 0.13	1.71 ± 0.38	9.91 ± 3.75	0.15 ± 0.03	36.32 ± 35.15

Section 6: 5-HT transporter/receptor gene analysis using qRT-PCR

Gene selection

Here, the overall aim was to investigate whether exposure to the tricyclic antidepressant (TCA) AMI interfered with the neurological pathways involved in the regulation of the serotonergic system. This system, as opposed to the adrenergic and dopaminergic systems, was selected as AMI is a tertiary amine TCA and therefore a more potent modulator of 5-HT as has been shown for imipramine [2]. Furthermore, both transporter and receptor proteins were selected for analysis, with the idea to capture at which point in the serotonergic signalling pathway, if any, the system was affected by AMI exposure. It was also important that selected genes had high sequence similarities to that in humans. The *slc6a4a* gene is homologous to the SERT gene in mammals and was therefore selected above its sister gene, *slc6a4b*, as it is more widely expressed in the zebrafish brain [3]. The *htr1aa* receptor gene was selected based upon several factors including its widespread expression in the zebrafish larva and adult brain [3], alongside its 76% similarity to the HTR1A receptor protein in mammals, whereas the other HTR1A homolog protein encoding gene, *htr1ab*, is only 68% similar [3]. Furthermore, it has been implicated in anxiety like states, where for example, mice lacking this receptor show increased anxiety-like behaviour [4], and rats administered with a HTR1A receptor agonist exhibit anxiolytic phenotypes [5]. Finally, the expression of *htr1aa* in zebrafish has been shown to be more sensitive to fluoxetine exposure compared to other 5-HT receptors [6]. The *htr2c* receptor gene was selected as it shares 54% similarity in its protein sequence with the human equivalent and it is also expressed in similar brain regions [7]. Furthermore, these genes have been reported in the literature to be up/down-regulated in fish following antidepressant

exposure. For example, all three genes have been found to be differentially expressed in zebrafish embryo-larvae following an 80-hour exposure to the SSRI FLX [8].

Primer selection and optimisation

Primer sequences specific for the target mRNA were taken from published studies [9,10] and purchased from Eurofins Genomics (Ebersberg, Germany). The optimal primer pair annealing temperature (T_a) for *hrt2c* was optimised by running a temperature gradient as Cunha *et al.* (2018), the source from which the target gene primer sequences was taken, did not explicitly state the optimum T_a [8]. The optimisation followed methods described previously [11]. PCR conditions included a denaturation step (heated to 95°C for 3 minutes), followed by 40 cycles of: denaturation at 95°C for 10 seconds, followed by annealing at each specific temperature in the gradient for 30 seconds (CFX96 Touch Real-Time PCR Detection System, Bio-rad). The highest temperature with the lowest CT value, and where replicates were most consistent, was selected as the optimal primer annealing temperature as this suggested the most efficient amplification. The T_a for the remaining three genes, *slc6a4a, htr1aa* and *rpl8* had already been established in our lab [9].

Supp Table 8. *Quantitative real-time PCR primer design and optimisation. GenBank (http://www.ncbi.nlm.nih.gov/) accession numbers, sense and antisense primer sequences, product size (base pairs, bp), annealing temperatures (T_a) and amplification efficiency (E) for the target genes studied: 5-HT transporter (slc6a4a), 5-HT receptors (hrt1aa and hrt2c) and the 'house keeping' gene (rpl8). The official zebrafish gene nomenclature guidelines were used to source target gene names (see http://www.zfin.org).*

**Target gene**	**GenBank accession number**	**Sense primer (5'-3')**	**Antisense primer (5'-3')**	**Product size (bp)**	**T_a (°C)**	**E**
slc6a4a	DQ285098	AGTGGACCTGGGCAATG	AGAAGATACGGCAAGAG AAG	81	57.5	1.93
hrt1aa	EH441641	AGAGCAGCGAGGTGAC	GAGCCGATGATTTGGTA AC	139	57.0	1.98
hrt2cl1	NM_001129 893.1	GCGCTCTCTGTCCTATTT GG	GTAGCGGTCGAGAGAAA TGG	89	57.0	2.03
rpl8	NM_200713	CCGAGACCAAGAAATCC AGAG	CCAGCAACAACACCAAC AAC	91	59.5	2.06

For each target gene, the linearity, detection range, amplification efficiency (*E*) and primer pair specificity was determined via a two-step amplification programme followed by a melt curve analysis. PCR reactions were run in triplicate across 5 dilutions of cDNA (a pool of cDNA from 8 samples from different treatments) starting with a 1:5 dilution, followed by a 10-fold dilution series (neat, 1:5, 1:50, 1:500 and 1:5000), alongside a no template control. PCR conditions included a denaturation step of 95°C for 3 minutes, followed by 40 cycles of: denaturation at 95°C for 10 seconds, followed by annealing at the optimised primer pair annealing temperature for 30 seconds. Melt curve analysis was then performed where products were denatured at 95°C for 1 minute and heated to 55°C for 1 minute, followed by 100 cycles lasting 5 seconds each beginning at 55°C with the temperature increasing 0.5°C each cycle. The specificity of primers was confirmed via a single peak in the melt curve. A summary of the optimised assay conditions is provided in supplementary table S4.

Real-time PCR quantification

Real time PCR was performed on a Real-time PCR Detection System (Bio-Rad) using the Bio-Rad CFX Maestro Software Version 2.2 (Bio-Rad). PCR reactions were run in triplicate in a 15 µl reaction mixture. Each gene was processed on two PCR plates (there were 56 samples in total, 8 from each of the 7 treatment groups) where samples were assigned to a plate location using a random number generator. Each plate therefore contained 28 samples in addition to the NTC and positive control (PC, a pool of cDNA from 8 samples from different treatments) to determine inter-assay variability. The PCR cycling and melt curve conditions were identical to those outlined above.

Section 7: Behaviour analysis

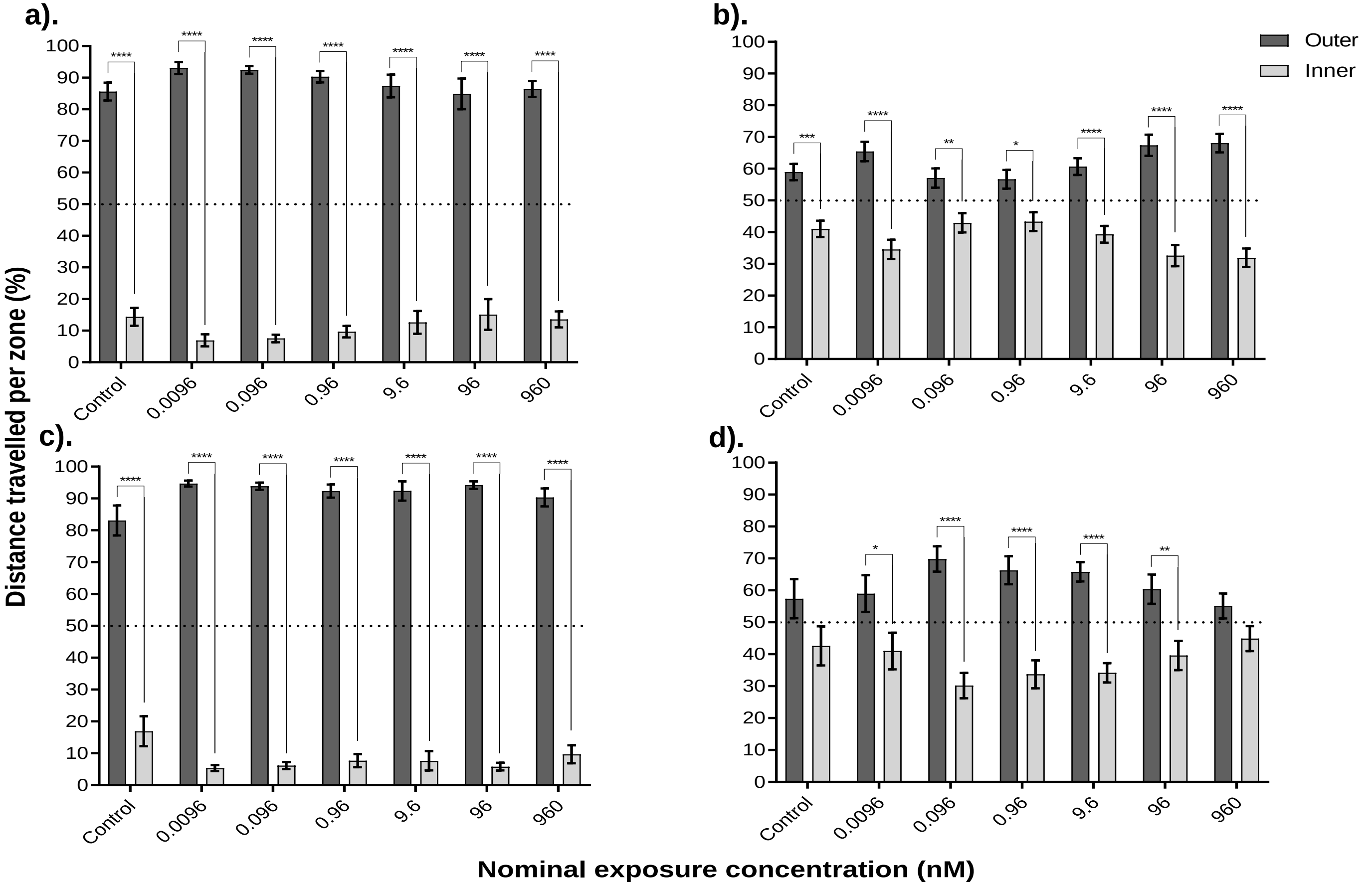

Supp Figure 5. *Pattern of fish zone preference displayed as the % total distance travelled in the outer versus the inner zone at 28 dpf during the (a) light and (b) dark phase and at 42 dpf during the (c) light and (d) dark phase (mean ± SEM, N = 16). Here, the dotted line represents equal travel across both areas. Differences in zone preference were assessed using Kruskal-Wallis and Dunn's Multiple Comparison tests, where significant differences are represented by * (P<0.05),** (P<0.01), *** (P<0.001) and **** (P<0.0001) versus the control treatment.*

Section 8: Reference list

(1) Carreño Gutiérrez, H.; O'Leary, A.; Freudenberg, F.; Fedele, G.; Wilkinson, R.; Markham, E.; van Eeden, F.; Reif, A.; Norton, W. H. J. Nitric Oxide Interacts with Monoamine Oxidase to Modulate Aggression and Anxiety-like Behaviour. *European Neuropsychopharmacology* **2020**, *30*, 30–43. https://doi.org/10.1016/j.euroneuro.2017.09.004.

(2) Fuxe, K.; Uncerstedt, U. Histochemical Studies on the Effect of (+)-Amphetamine, Drugs of the Imipramine Group and Tryptamine on Central Catecholamine and 5-Hydroxytryptamine Neurons after Intraventricular Injection of Catecholamines and 5-Hydroxytryptamine. *Eur J Pharmacol* **1968**, *4*, 135–144. https://doi.org/10.1016/0014-2999(68)90169-6.

(3) Norton, W. H. J.; Folchert, A.; Bally-Cuif, L. Comparative Analysis of Serotonin Receptor (HTR1A/HTR1B Families) and Transporter (Slc6a4a/b) Gene Expression in the Zebrafish Brain. *Journal of Comparative Neurology* **2008**, *511*, 521–542. https://doi.org/10.1002/cne.21831.

(4) Parks, C. L.; Robinson, P. S.; Sibille, E.; Shenk, T.; Toth, M. Increased Anxiety of Mice Lacking the Serotonin1A Receptor. *Proc Natl Acad Sci U S A* **1998**, *95*, 10734–10739. https://doi.org/10.1073/pnas.95.18.10734.

(5) Hernandes, P. M.; Batistela, M. F.; Vilela-Costa, H. H.; Sant'Ana, A. B.; Kumpel, V. D.; Tirapelle, M. C.; Bom, A. de O. P.; de Andrade, T. G. C. S.; Zangrossi, H. Role of 5-HT1A Receptors in the Ventral Hippocampus in the Regulation of Anxiety- and Panic-Related Defensive Behaviors in Rats. *Behavioural Brain Research* **2021**, *408* (April), 113296. https://doi.org/10.1016/j.bbr.2021.113296.

(6) Pei, S.; Liu, L.; Zhong, Z.; Wang, H.; Lin, S.; Shang, J. Risk of Prenatal Depression and Stress Treatment: Alteration on Serotonin System of Offspring through Exposure to Fluoxetine. *Sci Rep* **2016**, *6*, 33822. https://doi.org/10.1038/srep33822.

(7) Schneider, H.; Fritzky, L.; Williams, J.; Heumann, C.; Yochum, M.; Pattar, K.; Noppert, G.; Mock, V.; Hawley, E. Cloning and Expression of a Zebrafish 5-HT2C Receptor Gene. *Gene* **2012**, *502*, 108–117. https://doi.org/10.1016/j.gene.2012.03.070.

(8) Cunha, V.; Rodrigues, P.; Santos, M. M.; Moradas-Ferreira, P.; Ferreira, M. Fluoxetine Modulates the Transcription of Genes Involved in Serotonin, Dopamine and Adrenergic Signalling in Zebrafish Embryos. *Chemosphere* **2018**, *191*, 954–961. https://doi.org/10.1016/j.chemosphere.2017.10.100.

(9) Filby, A. L.; Paull, G. C.; Hickmore, T. F. A.; Tyler, C. R. Unravelling the Neurophysiological Basis of Aggression in a Fish Model. *BMC Genomics* **2010**, *11* (1). https://doi.org/10.1186/1471-2164-11-498.

(10) Cunha, V.; Rodrigues, P.; Santos, M. M.; Moradas-Ferreira, P.; Ferreira, M. Fluoxetine Modulates the Transcription of Genes Involved in Serotonin, Dopamine and Adrenergic Signalling in Zebrafish Embryos. *Chemosphere* **2018**, *191*, 954–961. https://doi.org/10.1016/j.chemosphere.2017.10.100.

(11) Filby, A. L.; Tyler, C. R. Appropriate "housekeeping" Genes for Use in Expression Profiling the Effects of Environmental Estrogens in Fish. *BMC Mol Biol* **2007**, *8*. https://doi.org/10.1186/1471-2199-8-10.
